# Brain clearance is reduced during sleep and anesthesia

**DOI:** 10.1038/s41593-024-01638-y

**Published:** 2024-05-13

**Authors:** Andawei Miao, Tianyuan Luo, Bryan Hsieh, Christopher J. Edge, Morgan Gridley, Ryan Tak Chun Wong, Timothy G. Constandinou, William Wisden, Nicholas P. Franks

**Affiliations:** 1 Department of Life Sciences, Imperial College London, South Kensington, London, UK; 2 UK Dementia Research Institute, Imperial College London, London, UK; 3 Centre for Doctoral Training and Centre for Neurotechnology, Imperial College London, London, UK; 4 Department of Electrical and Electronic Engineering and UK Dementia Research Institute, Care Research & Technology, Imperial College London, London, UK; 5 Present Address: Department of Anesthesiology, Affiliated Hospital of Zunyi Medical University, Zunyi, China; 6 Present Address: Guizhou Key Laboratory of Anesthesia and Organ Protection, Zunyi Medical University, Zunyi, China

**Keywords:** Sleep, Biophysics

## Abstract

It has been suggested that the function of sleep is to actively clear metabolites and toxins from the brain. Enhanced clearance is also said to occur during anesthesia. Here, we measure clearance and movement of fluorescent molecules in the brains of male mice and show that movement is, in fact, independent of sleep and wake or anesthesia. Moreover, we show that brain clearance is markedly reduced, not increased, during sleep and anesthesia.

## Abstract (web summary)

It has been widely believed that a key function of sleep is to actively clear metabolites and toxins from the brain. Miao, Luo et al. show in mice that brain clearance is markedly reduced—not increased—during sleep and anesthesia.

## Main

Sleep is a state of vulnerable inactivity. Because of the risks that this vulnerability entails, most researchers assume that sleep must confer some essential benefit^1–3^. However, what this is remains a mystery. One suggestion is that sleep clears the brain of metabolites and toxins using the ‘glymphatic’ system, a process that cannot operate efficiently during the waking state^3,4^. This attractive idea has important implications. For example, diminished toxin clearance brought about by chronically poor sleep might exacerbate, if not cause, Alzheimer disease^5,6^.

How metabolites and toxins are cleared from the brain is unresolved. Disputes surround both the anatomical pathways^7–9^ and the mechanisms of clearance^7,10,11^. The glymphatic hypothesis contends that bulk flow of fluid, rather than just diffusion, actively clears solutes from the brain parenchyma during non-rapid-eye-movement (NREM) sleep^3^. This flow is proposed to be driven by hydrostatic pressure gradients established by arterial pulsations^12^. Anesthetics at sedative doses, which induce states resembling deep NREM sleep^2,13^, were also reported to increase clearance^3,14,15^. However, whether sleep does enhance clearance by increased bulk flow is unresolved, with findings both supporting^3,4,12,14–16^ and challenging^10,11,17–19^ the idea. Here, we directly measure clearance and fluid movement in the brains of mice during different vigilance states (awake, sleeping or sedated).

**Fig. 1:**
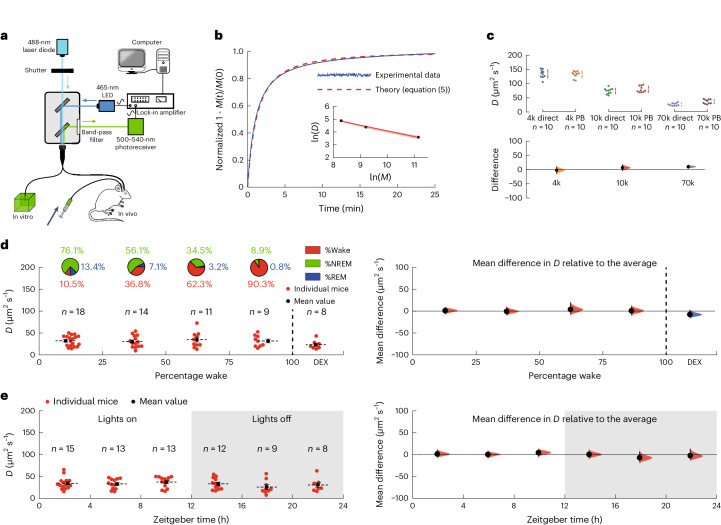
Changes in local diffusion with vigilance states. **a**, The experimental setup. Light from a 488-nm laser diode was passed through a 200-μm optical fiber into either an agarose gel brain phantom in vitro or the frontal cortex of a mouse in vivo. For the in vitro experiments, the agarose gel contained 4 kDa FITC-dextran while, for the in vivo experiments, the brain had been injected with 4 kDa FITC-dextran some hours earlier. **b**, A typical recording of photobleaching in an agarose gel brain phantom, fitted by least-squares to equation (5), to give (for this example) a value of *D* = 136 μm^2^ s^−1^. The inset shows that the diffusion coefficient follows a power law, with *D* ∝ *M*^−0.44^. The red shading in the inset shows the s.e.m. **c**, A comparison between the diffusion coefficients determined directly (direct) (Methods and Extended Data Fig. 3) and those determined using the photobleaching method (PB) was not significantly different (two-way ANOVA *P* = 0.10). Top, the individual data points. Bottom, the differences in the diffusion coefficients determined using the two methods. The agreement between the methods was excellent at 4 kDa FITC-dextran and this was used for the in vivo measurements. **d**, Left, the diffusion coefficients of 4 kDa FITC-dextran as a function of the percentage of wake (state) during the hour the diffusion coefficient was being measured (the distribution of vigilance states is shown in the pie charts above). Each point represents the average of typically four measurements for an individual mouse and the number of mice, *n*, is shown above. The last group of data on the right-hand side were recorded during dexmedetomidine (DEX) sedation. Right, the mean differences relative to the average diffusion coefficient across all vigilance states. A one-way ANOVA gave *F*(4,55) = 0.90; *P* = 0.47. (A difference of ~35% in *D* would have been detected.) **e**, Left, the diffusion coefficients as a function of zeitgeber time. Right, the mean differences relative to the average diffusion coefficient recorded over the circadian cycle. A one-way ANOVA gave *F*(5,64) = 0.88; *P* = 0.50. In **c**–**e**, the vertical solid lines show the 95% confidence intervals; the shaded areas show the distributions of likelihood. In **d** and **e**, the horizontal solid and dashed lines show the s.e.m. and the mean, respectively. Source data

We first determined the diffusion coefficient (*D*) of a fluorescent dye (fluorescein isothiocyanate, FITC-dextran) in brains of mice (Fig. 1a). We injected 4 kDa FITC-dextran into the caudate putamen (CPu) and then monitored the fluorescence arriving in the frontal cortex. The first series of experiments involved waiting for steady state and then bleaching the dye in a small volume of tissue in the neocortex and determining *D* from the rate that unbleached dye moved into the bleached region, a technique pioneered by others^20,21^.

**Extended Data Fig. 1:**
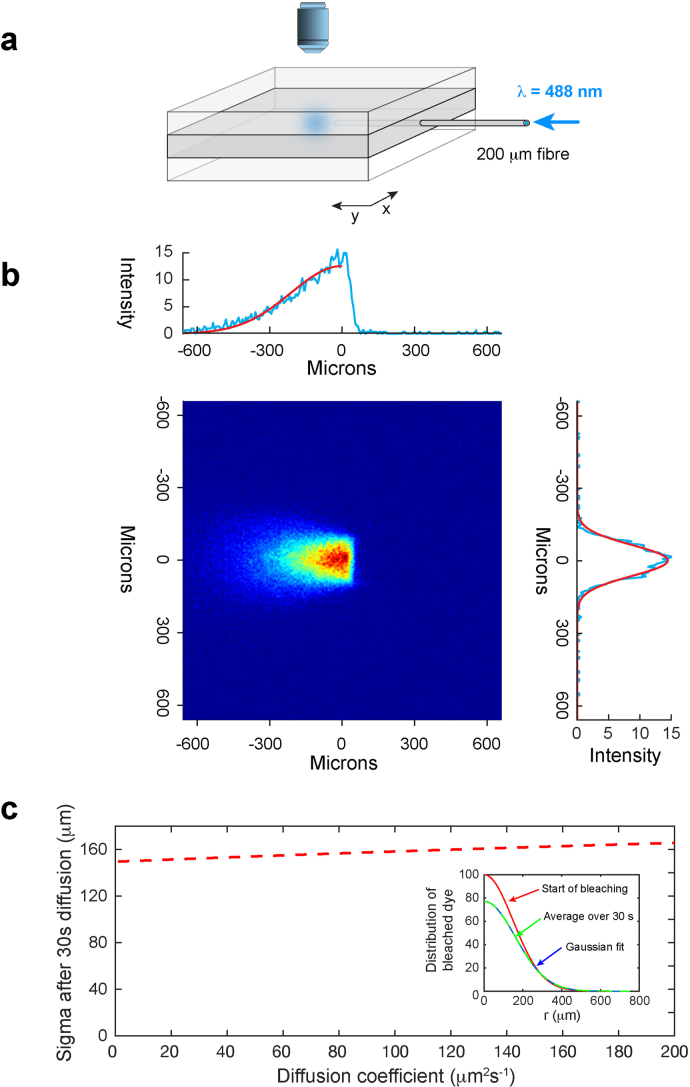
Measurement of the distribution of bleached dye. **a**, A thin brain slice or sheet of an agarose gel brain phantom containing FITC-dextran was sandwiched between two blocks of clear agarose (Methods). An optical fiber (core diameter 200 μm) was inserted into the brain slice or central gel and an image taken of the light distribution of a 488 nm-laser diode. **b**, The intensity distribution from a digitized image (blue lines) was then fit to a hemispherical Gaussian distribution (red solid lines). The average value for the standard deviations of the Gaussian fits was *σ* = 149.5 μm (CI [140.7, 162.6]; *n* = 8 independent experiments). **c**, There is a small change in this standard deviation due to diffusion during the 30 s of bleaching (red dashed line), which differs for each molecular weight due to the different diffusion coefficients. This was estimated by averaging the dye distribution (equation [2]) over 30 s and then fitting this to a Gaussian. Inset: An example of how the dye distribution changes during bleaching for 4 kDa FITC-dextran, *D* = 133.9 μm^2^ s^−1^. The red curve is the Gaussian distribution at the start of bleaching (*σ* = 149.5 μm), the green curve is the average distribution over 30 s, fitted to a Gaussian (blue dashed line) which gives (*σ* = 161.0 μm).). The values of *σ* that were used for the diffusion measurements in agarose gel for 4 kDa, 10 kDa and 70 kDa FITC-dextran were 152.1 μm, (CI [143.3, 165.0]; *n* = 8 independent experiments), 156.2 μm, (CI [147.7, 169.0]; *n* = 8 independent experiments) and 161.0 μm, (CI [152.6, 173.2]; *n* = 8 independent experiments), respectively. Source data

**Extended Data Fig. 2:**
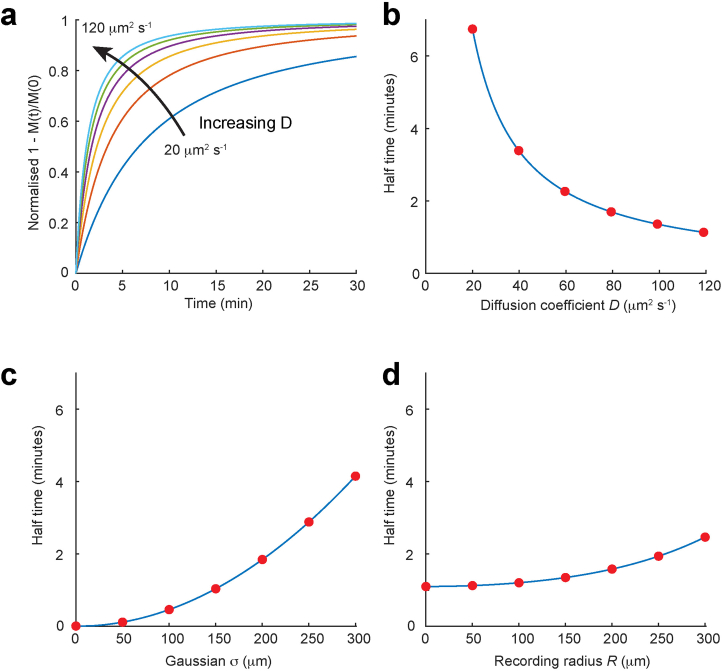
The time course of *M*(*t*) (equation [5]) is sensitive to values of *D* and *σ*, but insensitive to values of *R.* **a**, The time course of *M*(*t*)/*M*(0) for values of *D* from 20–120 μm^2^ s^−1^. **b**, Corresponding half times of *M*(*t*)/*M*(0) over the same range of *D* showing that the half times change greatly with *D*. **c**, Corresponding half times of *M*(*t*)/*M*(0) over the same range of *D* showing that the half times change greatly with *σ*. **d**, Corresponding half times of *M*(*t*)/*M*(0) over the same range of *D* showing that the half times change little with *R*. **Derivation of equation** [5]. The total number of mols *M(t)* of fluorescent dye in a hemisphere of radius R, is given by equation [2] multiplied by the area of a hemisphere (2*πr*^2^), integrated from 0→*R* (because we have assumed that the volume being recorded from is a hemisphere of radius *R)*: $M(t)=C(0,0){[1+\frac{2Dt}{{\sigma }^{2}}]}^{-\frac{3}{2}}\underset{0}{\overset{R}{\int }}2\pi {r}^{2}exp[\frac{-{r}^{2}}{4Dt+2{\sigma }^{2}}]dr$ This can be written as: $M(t)=a\underset{0}{\overset{R}{\int }}{r}^{2}exp[-b{r}^{2}]dr$, where $a=2\pi C(0,0){[1+\frac{2Dt}{{\sigma }^{2}}]}^{-\frac{3}{2}}$ and $b={(4Dt+2{\sigma }^{2})}^{-1}$ Integrating by parts gives: $M(t)=[-\frac{aR}{2b}exp[-b{R}^{2}]]+\underset{0}{\overset{R}{\int }}\frac{a}{2b}exp[-b{r}^{2}]dr$ Using the standard integral: $\underset{0}{\overset{R}{\int }}exp[-b{r}^{2}]dr=\sqrt{\frac{\pi }{4b}}erf(\sqrt{b}R)$, we have $M(t)=\frac{a}{2b}\{\sqrt{\frac{\pi }{4b}}erf(\sqrt{b}R)-Rexp[-b{R}^{2}]\}$ so, finally, substituting in *a* and *b* we have Equation [5]: $M(t)=\frac{2\pi C(0,0){\sigma }^{3}}{\sqrt{(2Dt+{\sigma }^{2})}}\{\sqrt{\frac{\pi (2Dt+{\sigma }^{2})}{2}}erf(\frac{R}{\sqrt{(4Dt+2{\sigma }^{2})}})-Rexp[-\frac{{R}^{2}}{(4Dt+2{\sigma }^{2})}]\}$.

We validated our methodology by measuring the diffusion of FITC–dextrans of various molecular weights in agarose ‘brain phantom’ gels, modified to approximate the light-scattering and optical-absorption properties of brain tissue^22^ and found (Extended Data Fig. 1) that the distribution of light intensity was well approximated by a hemispherical Gaussian distribution. Immediately following 30 s of bleaching, we recorded the recovery of the fluorescence as unbleached dye moved into the bleached volume. Figure 1b shows a typical recording for 4 kDa FITC-dextran (blue trace). There was excellent agreement between these data and the time course predicted using equations (4) and (5) (Methods and Extended Data Fig. 2).

**Extended Data Fig. 3:**
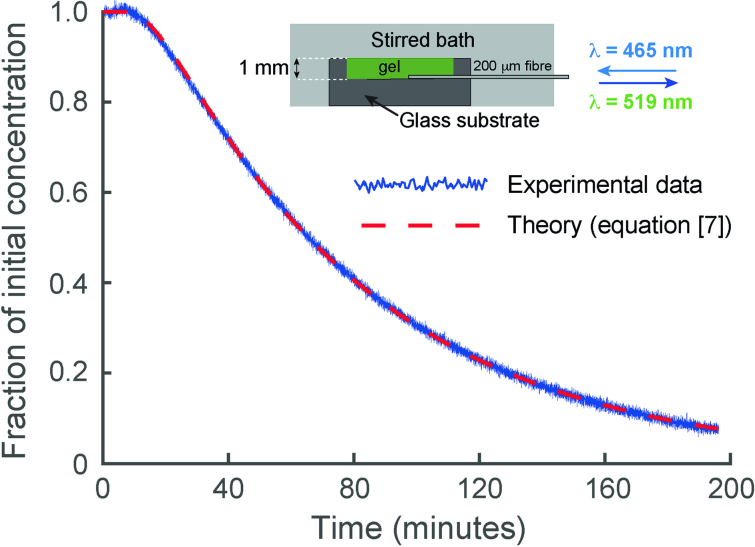
Direct measurement of diffusion coefficients. The diffusion coefficients of the FITC–dextrans in the brain phantom agarose gel were determined directly by measuring the time course of diffusion of FITC-dextran from a 1-mm thick sheet of gel, into an effectively infinite stirred water bath containing all the components of the brain phantom (except the agarose and FITC-dextran). By recording the reduction in the fluorescent signal close to the impermeable glass surface on which the gel was set, as a function of time, the diffusion coefficient could be directly determined using equation [7] (ref. ^26^). The figure shows data from a typical experiment using 4 kDa FITC-dextran (blue trace) and the red dashed line shows the change predicted by equation [7]. Source data

Using this method, our measured diffusion coefficients were in good agreement with literature values in aqueous solutions^23,24^ and their mass dependence (inset to Fig. 1b). Our diffusion coefficients also agreed well (Fig. 1c) with values obtained using a direct method (Extended Data Fig. 3) that did not involve photobleaching.

**Extended Data Fig. 4:**
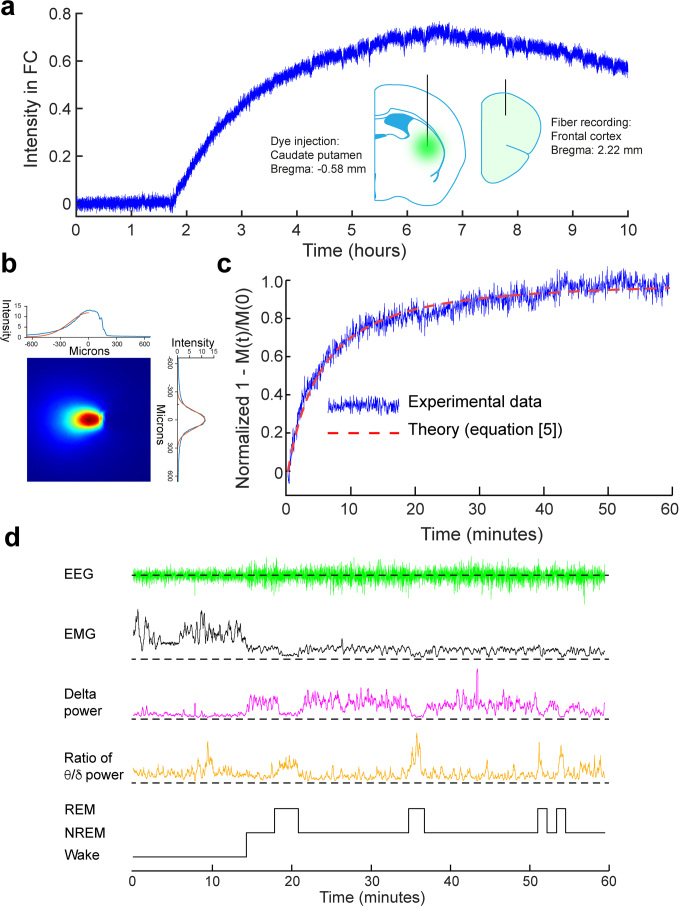
Measurement of movement *in vivo* using photobleaching. **a**, Fluorescent intensity measured in the frontal cortex following injection of 4 kDa FITC-dextran into the CPu (at *t* = 0). After a delay, fluorescent intensity rises to a maximum and then slowly decays. **b**, As with the experiments in gels, the spread of light in the brain had to be established. This was done using brain slices (Methods) and this figure shows a typical image obtained from a brain slice, which provided a measure of the standard deviation of the hemispherical gaussian σ. **c**, A typical recording *in vivo* of the recovery of fluorescence after photobleaching. A value for *D* was derived from the theoretical fit (red dashed line) to Eq. 5, as described in Methods. **d**, Throughout the experiment, the EEG and EMG signals were recorded and the power in the delta band (1–4 Hz) and theta band (5–10 Hz) derived, so that the vigilance state (WAKE, NREM or REM) could be determined (Methods). Source data

We then measured *D* in vivo using 4 kDa FITC-dextran, which after injection into the CPu, could be detected in the frontal cortex, where its fluorescence peaked at about 6–7 h postinjection, then slowly declined at ~6% per hour (Extended Data Fig. 4a). During the slowly declining phase, approximating to steady state, the recovery from bleaching was recorded (and baseline corrected) (Methods). The spread of light in a brain using a brain slice (Methods) confirmed that the distribution was also well approximated by a hemispherical Gaussian distribution (Extended Data Fig. 4b). As with the gel experiments described above, the fluorescence recovery agreed well with the theoretical predictions (Extended Data Fig. 4c) and we derived values for the effective tissue *D* from the time courses, while also determining the vigilance states (Extended Data Fig. 4d).

**Extended Data Fig. 5:**
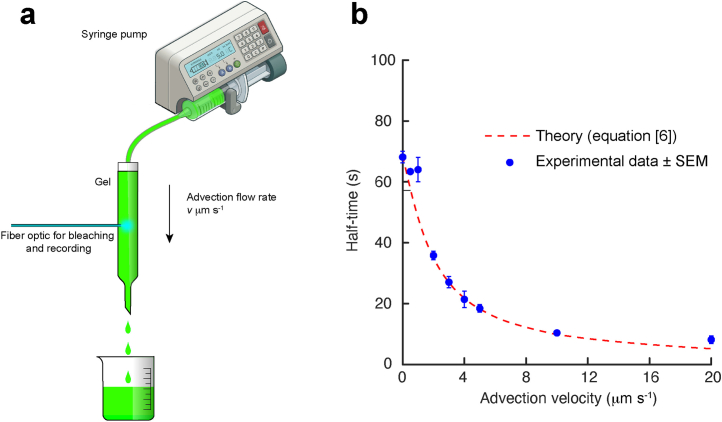
The effect of advective flow on the time course of recovery of fluorescence after photobleaching. This was assessed in an *in vitro* experiment illustrated in **a**. A solution of 4 kDa FITC-dextran was passed through a gel (Methods) at a constant flow rate and the time course for the recovery of photobleaching recorded using an optical fiber, exactly as used in the experiments described in the text *in vitro* and *in vivo*. **b**, The observed half times were accurately predicted from equation [6] and reduced rapidly with increasing advective velocity. From the precision with which we could record changes in diffusion coefficients *in vivo* (Fig. 1d,e right panels) and their corresponding half times, we estimate that we would have been able to detect a change in advective flow of about 0.5 μm/s, or greater. Where error bars (SEM; *n* = 5 independent experiments) are not shown they were smaller than the size of the symbol. Source data

We observed no significant change in the diffusion coefficient of 4 kDa FITC-dextran with either vigilance state or dexmedetomidine (200 μg kg^−1^; intraperitoneal (i.p.) sedation (Fig. 1d) or during the day–night cycle (Fig. 1e)). The mean value for *D* across all vigilance states was 32.1 ± 1.9 μm^2^ s^−1^ (*n* = 52; mean ± s.e.m.), which corresponds, using equation (3), to a tortuosity of ~2.5 (having corrected the aqueous *D* to 37 °C using the Stokes–Einstein equation^25^). This is consistent with values reported for rodent neocortex^25^ and suggests that the movement of 4 kDa FITC-dextran in the cortex is predominantly by diffusion, a conclusion previously reached by others^11,18,19^. Notably, these results show that diffusion kinetics do not change during sleep or anesthesia. From separate in vitro measurements (Extended Data Fig. 5), we estimate that we could have detected a change in bulk flow between vigilance states of >0.5 μm s^−1^ but our results cannot rule out changes in pairwise flows in opposite directions over small distances in the surrounding tissue, which might have averaged out, so that brain clearance might, nonetheless, have changed. We therefore extended our experiments to measure brain clearance itself during different vigilance states.

**Fig. 2:**
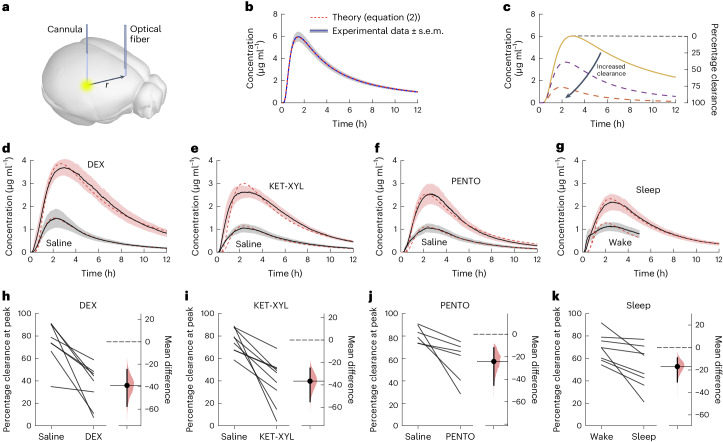
Photometry data show that brain clearance is reduced by sleep and anesthesia. **a**, A fluorescent dye (AF488) was injected into the CPu and the fluorescence monitored over time in the frontal cortex. **b**, The spread of the dye could be accurately predicted by equation (2) in an agarose gel with a diffusion coefficient of 295 μm^2^ s^−1^, where there was zero clearance. The error envelope represents the s.e.m. **c**, If brain clearance of the dye is assumed to increase with time as described by equation (9), then the concentration in the frontal cortex is predicted to follow the time course given by equation (8) and is shown by the dashed lines. Knowing the concentration that should have arrived at the cortex had there been no clearance (solid line), the percentage clearance can be calculated at any time. **d**–**g**, Observed concentration curves recorded following either saline injection or DEX anesthesia (**d**), KET-XYL anesthesia (**e**), PENTO anesthesia (**f**) and during the waking state or during sleep (**g**). The observed concentrations were significantly lower (two-way ANOVA with Bonferroni–Holm multiple comparisons correction) in the waking state compared to DEX (*P* < 10^−6^), ketamine-xylazine (KET-XYL) (*P* < 10^−6^) or pentobarbital (PENTO) (*P* < 10^−6^) anesthesia or during sleep (*P* < 10^−6^). The error envelopes represent the s.e.m. **h**–**k**, Peak clearance observed following either saline injection or DEX anesthesia (**h**), KET-XYL anesthesia (**i**), PENTO anesthesia (**j**) and during the waking state or during sleep (**k**). For both anesthesia and sleep, the percentage of brain clearance was significantly reduced (two-tailed paired *t*-test): DEX (*P* = 0.0029), KET-XYL (*P* = 0.0015) or PENTO (*P* = 0.037) anesthesia or during sleep (*P* = 0.016). The vertical bars represent 95% confidence intervals about the mean (horizontal solid lines) and the shaded areas are the distributions of likelihood. Source data

**Extended Data Fig. 6:**
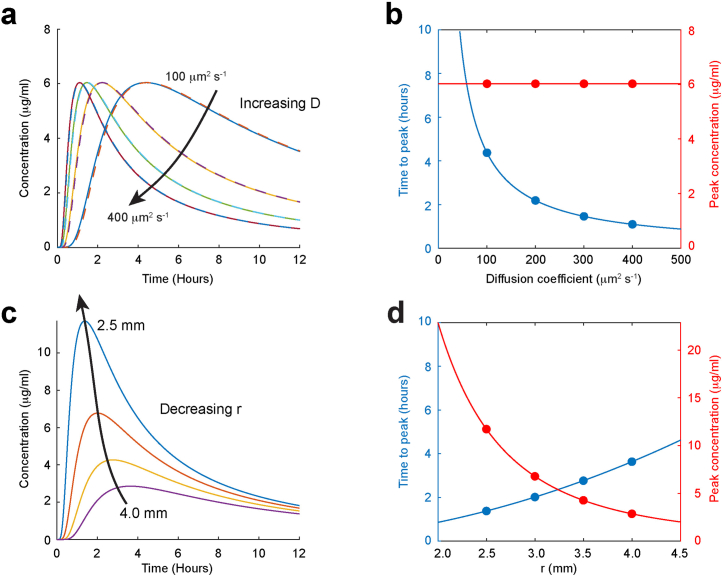
The time course of C(r, t). **a**, **b** According to equation [2], the concentration at a fixed distance, *r*, from a Gaussian source (solid lines) reaches a peak with time that depends only on the diffusion coefficient *D*, while the peak concentration does not change. Almost identical concentrations are predicted if the source is a sphere, rather than a Gaussian, containing the same number of moles (dashed lines). (The equation for the concentration as a function of time from a spherical source has been solved by Crank^26^.) **c,d**, The peak concentration with time for a fixed diffusion coefficient, decreases with increasing distance *r*, with relatively small changes in the time to peak.

The approach we took to measuring brain clearance used the same experimental setup as shown in Fig. 1a. However, it has recently been shown^16^ that a small dye which moves freely in the parenchyma can be used to accurately quantify brain clearance (Fig. 2a). This would also allow a complete time course to be recorded in the cortex as the dye spread throughout the brain. We used AF488 (~570 Da) and first showed that the spread in a gel, with no clearance possible, could be accounted for by equation (2), the spread from a Gaussian source. Figure 2b, shows that equation (2) fitted the experimental data essentially perfectly, with an aqueous diffusion coefficient of 295 μm^2^ s^−1^. In the absence of clearance and, if *r* (the distance between where dye is injected and where it is recorded) is constant, then the timing of the peak is determined only by the diffusion coefficient (Extended Data Fig. 6). If clearance occurs, the height of the peak would be reduced (Fig. 2c and equation (8)).

**Extended Data Fig. 7:**
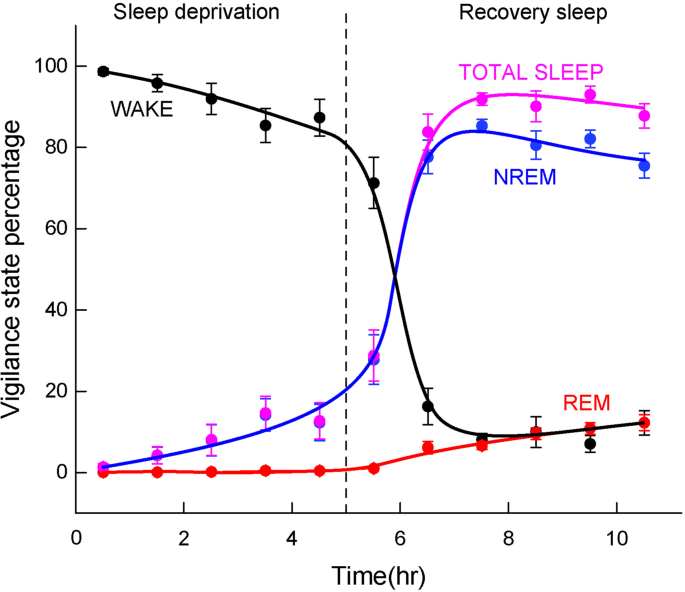
Vigilance-state percentages for the sleep photometry experiments. During the sleep experiments, mice were first sleep deprived by placing novel objects in their cage and after 5 hours were then allowed to sleep. The photometry measurements during the WAKE state were carried out during the five hours of sleep deprivation, where the WAKE state occurred, on average, 92% of the time (8% NREM and 0% REM). The photometry measurements during the SLEEP state were carried out after the first sleep episode following sleep deprivation. During the first five hours the vigilance state percentages were: WAKE 9.3% (*n* = 11 mice), NREM 80.8% (*n* = 13 mice), REM 9.9% (*n* = 13 mice), TOTAL SLEEP 90.7% (*n* = 13 mice). Sleep scoring of vigilance states was carried out as described in Methods. The errors bars represent SEMs. Source data

**Extended Data Fig. 8:**
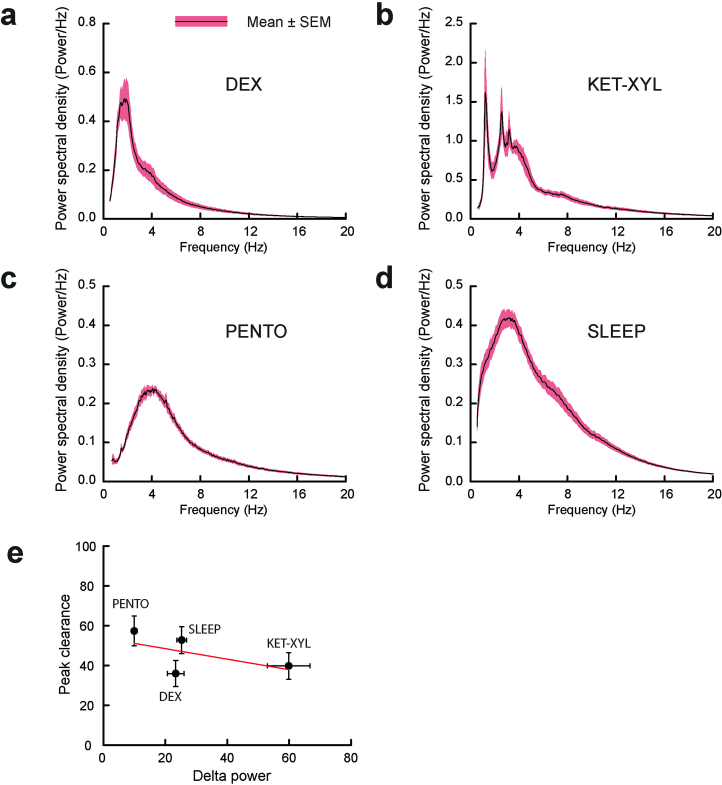
Power spectra during anesthesia and correlation with peak clearance. Power spectral density plots during anesthesia were calculated for the three anesthetics **a-c** and **d**, during recovery sleep. For the anesthetics, the power spectra were carried out using the EEG recorded during the first 2 hours of anesthesia (excluding the first ten minutes following injection). For sleep, the power spectra were calculated during 2 hours of recovery sleep, which included some time in WAKE (9.3%) and REM (9.9%) states. **e**, There was a weak negative correlation (Pearson’s correlation coefficient −0.58) between delta (0.5–4.0 Hz) power and peak clearance (see Fig. 2d–g and Extended Data Table 1). PENTO (*n* = 10 mice), DEX (*n* = 9 mice), SLEEP (*n* = 9 mice) and KET/XYL (*n* = 9 mice). The errors bars represent SEMs and where they are not shown they were smaller than the size of the symbol. Source data

We then repeated these experiments in mice which had been injected (i.p.) with either saline or an anesthetic (Fig. 2d–f). A comparison was also made between the sleeping and waking states (Fig. 2g). For the saline controls, the peak concentrations were much lower than that predicted by equation (2) but could be accounted for accurately by assuming clearance had occurred, as described by equations (8) and (9). There was excellent agreement between the photometry data and equation (8), with the discrepancies at small times possibly being due to dye finding its way across the brain via the ventricles^16^. At the peak concentration (~2–3 h) the clearance was 70–80% with saline-injected controls, indicating that the normal mechanisms of brain clearance had not been disrupted. Notably, in the presence of anesthetics, this clearance was substantially reduced. This was true for dexmedetomidine (Fig. 2d,h), ketamine-xylazine (Fig. 2e,i) and pentobarbital (Fig. 2f,j). Reduced clearance was also observed in mice that were sleeping, compared with mice that were kept awake (Fig. 2g,k and Extended Data Fig. 7). By contrast, the diffusion coefficients, reflecting the rate of spread in the brain parenchyma and the time to reach the peak in the photometry data (Fig. 2d–g), did not change significantly during sleep or anesthesia (Extended Data Table 1). If these diffusion coefficients reflect pure diffusion, then they would correspond to a tortuosity of ~1.4. We cannot rule out that spread might be enhanced by local fluid movement without bulk flow; however, these do not change with vigilance state. We also measured the EEG power spectra (Extended Data Fig. 8a–d) and found a weak negative correlation between peak clearance and delta (0.5–4 Hz) power (Extended Data Fig. 8e), implying that the deeper the sleep, the lower the clearance.

**Fig. 3:**
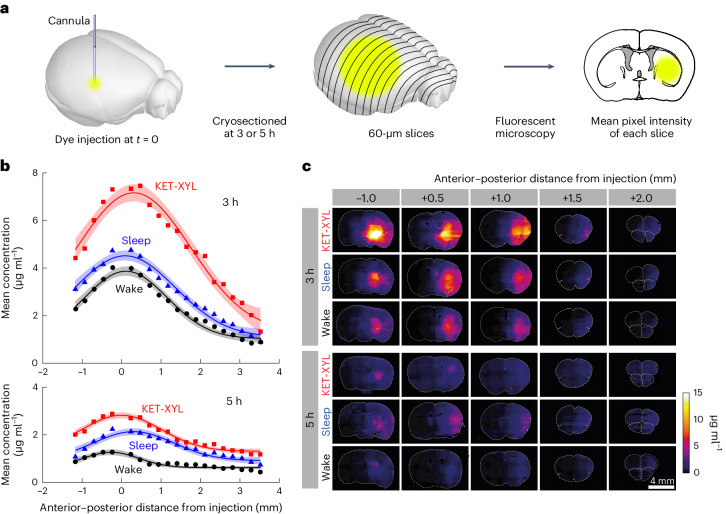
Histology data confirm that brain clearance is reduced by sleep and anesthesia. **a**, At either 3 or 5 h following injection of AF488 into the CPu, the brain was frozen and cryosectioned at 60 μm. The average fluorescent intensity across each slice was obtained by fluorescent microscopy; then the mean intensities across groups of four slices were averaged. **b**, The mean fluorescence intensity was converted to a concentration using the calibration data in Supplementary Fig. 1 plotted against the anterior–posterior distance from the point of injection for wake (black), sleep (blue) and KET-XYL (red) anesthesia. Top, the data after 3 h. Bottom, the data after 5 h. The lines are Gaussian fits to the data and the error envelopes show the 95% confidence intervals. At both 3 and 5 h, the concentrations during KET-XYL (*P* < 10^−6^ at 3 h; *P* < 10^−6^ at 5 h) and sleep (*P* = 0.0016 at 3 h; *P* < 10^−4^ at 5 h) were significantly larger than wake (two-way ANOVA with Bonferroni–Holm multiple comparisons correction). **c**, Representative images of the brain slices across the brain (anterior–posterior distance from the site of AF488 injection) at both 3 h (top three rows) and 5 h (bottom three rows). Each row represents data for the three vigilance states (wake, sleep and KET-XYL anesthesia). The color scale on the right shows the concentrations, determined using the calibration data in Supplementary Fig. 1. Source data

Histology experiments (Fig. 3) confirmed the photometry results. At both 3 h (Fig. 3b, top) and 5 h (Fig. 3b, bottom) after dye injection, the concentration of dye was higher during sleep and ketamine-xylazine anesthesia. As expected, (equation (8)), the spread was Gaussian (fitted curves in Fig. 3b), with characteristic widths roughly in line with those predicted using the diffusion coefficients derived from the photometry experiments. These data show that redistribution of the AF488 dye is essentially by diffusion alone and confirm that sleep and ketamine-xylazine anesthesia inhibit clearance. Representative brain sections are shown in Fig. 3c at 3 h (top) and 5 h (bottom).

Our experiments show that brain clearance is reduced during sleep and anesthesia, the opposite conclusion of ref. ^3^. Those authors observed that fluorescent dyes injected into the cerebrospinal fluid (CSF) via the cisterna magna penetrated further into the cortex during sleep and anesthesia. They interpreted this as showing that molecular movement into the cortex must be faster during these states. However, the concentration of dye in any brain region will always be the difference between its rate of arrival and its rate of departure and so increased dye penetration in sleep and anesthesia can be equally well explained by a reduced rate of clearance rather than an increased rate of entry. Indeed, almost all the experiments that have been interpreted as showing that sleep or anesthesia change brain clearance have involved introducing markers into the CSF, which then move into the brain parenchyma^14,26–30^. Under these circumstances, entry, exit and redistribution of the marker are all occurring simultaneously, greatly confounding any quantification of clearance.

**Extended Data Fig. 9:**
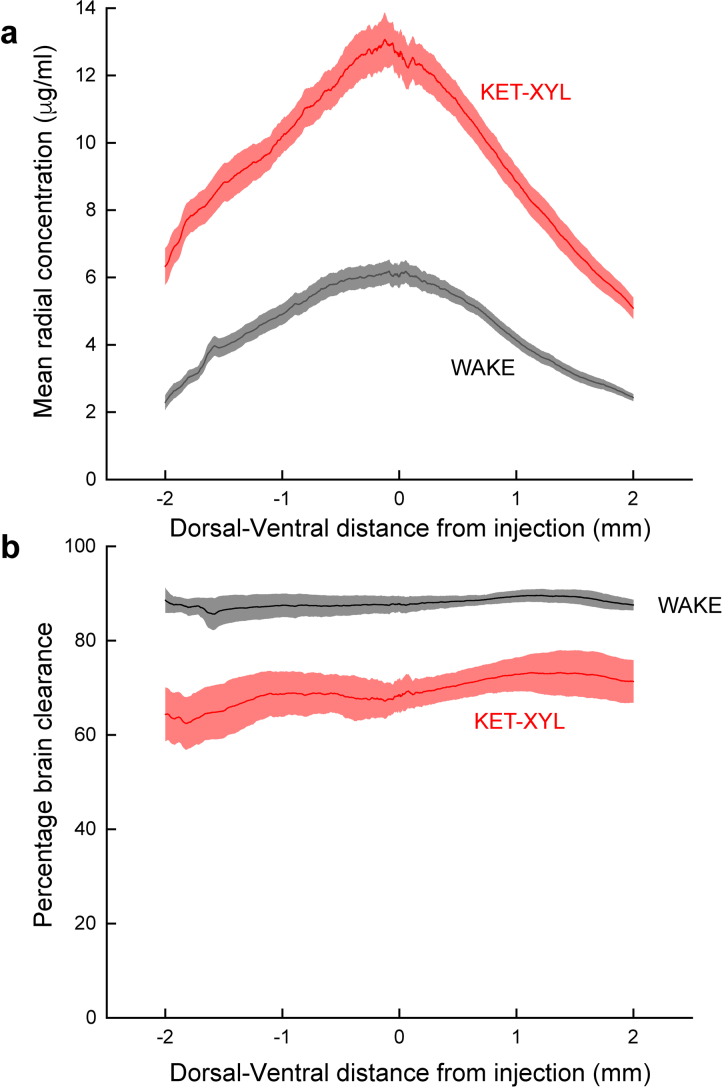
Brain clearance is uniform across the brain. The concentration of AF488 dye 3 hours after injection into the CPu was measured at an anterior-posterior coordinate 1 mm from the site of injection. **a**, The concentration of dye was then calculated as a function of radial distance from the peak concentration in both the dorsal and ventral directions. As predicted by equation [8], this results in a Gaussian curve. **b**, Using these data (predicted by equation [8]) together with equation [2], the percentage clearance can be calculated in the dorsal and ventral directions. Two-way ANOVA shows that there is no significant change in brain clearance across the brain (*p* = 0.99) for both WAKE animals and those anesthetized by ketamine-xylazine. In contrast, the inhibition of clearance by ketamine-xylazine is highly significant (*p* < 10^−6^). For both panels the means are for *n* = 3 animals and the error envelope shows the SEMs. Source data

Our data in Figs. 2 and 3 show that, averaged across the brain, clearance is reduced by both sleep and anesthesia. Although clearance might vary with anatomical location, the extent of this variation appears small (Extended Data Fig. 9). Moreover, the inhibition of clearance by ketamine-xylazine is highly significant independent of location. These data are for a small dye that can freely move in extracellular space. Molecules of larger molecular weights may behave differently. Exactly how anesthetics and sleep inhibit brain clearance is unclear, although it is notable that CSF outflow from the brain is markedly reduced by anesthetics^30^. Whatever the mechanism, however, our results challenge the idea that the core function of sleep is to clear toxins from the brain.

## Methods

### Theoretical basis of three-dimensional photobleaching method

We assume that, following bright illumination, the bleached fluorescent dye is distributed over a hemispherical volume with a concentration, *Q*(*s*), that falls off as a Gaussian distribution (see main text and Extended Data Figs. 1b and 4b for experimental confirmation):

1 $$Q(s)=Q(0){\rm{exp}}\left(-\frac{{s}^{2}}{2{\sigma }^{2}}\right)(s\ge 0)$$

where *Q*(0) is the maximum tissue concentration of the bleached dye at the origin of the hemisphere, *s* is the radial distance from the center of the distribution and *σ* is the standard deviation of the Gaussian distribution. Then, following bleaching, the concentration *C*(*r*,*t*) of bleached dye as a function of time, *t*, and distance, *r*, from the center of the hemisphere can be shown to be:

2 $$C(r,t)=C(0,0)\left\{{\left[1+\frac{2Dt}{{\sigma }^{2}}\right]}^{-\frac{3}{2}}\left[{\rm{exp}}\left(\frac{-{r}^{2}}{4Dt+2{\sigma }^{2}}\right)\right]\right\},$$

where *D* is the effective diffusion coefficient governing movement through the tissue. (This result was originally obtained^31^ for the case of a spherical ‘volume source’ in the atmosphere and the subsequent diffusion of material from the source.) The effective diffusion coefficient, *D*, through the tissue is related to the aqueous diffusion coefficient, *D*_aq_, by

3 $$D={D}_{\rm{aq}}/{\lambda }^{2}$$

where the dimensionless parameter *λ* is the empirical tortuosity, which accounts for the resistance to diffusion and increased path length which a membrane-impermeable dye encounters when diffusing through the tortuous extracellular space^32^.

The fluorescent signal *I*(*t*) which is recorded at any time *t* after bleaching is due to unbleached dye diffusing back into the bleached volume. If we assume the volume being recorded from is a hemispherical volume of radius *R* and that *I*(0) is the signal recorded immediately after bleaching (at *t* = 0) and *I*(∞) is the signal recorded when equilibrium has been re-established (which is also the signal recorded immediately before bleaching), then *M*(*t*), the number of moles of bleached dye in the hemispherical volume at a time *t*, is related to the observed fluorescent intensities by:

4 $$M(t)=M(0)\left[\frac{I(\infty )-I(t)}{I(\infty )-I(0)}\right]$$

where *M*(0) is the number of moles of bleached dye in the hemisphere immediately following bleaching.

The total number of moles *M*(*t*) of fluorescent dye in a hemisphere of radius *R*, is given by equation (2) multiplied by the area of a hemisphere (2π*r*^2^), integrated from 0→*R*, which leads to (Extended Data Fig. 2):

5 $$\begin{array}{l}M(t)=\displaystyle\frac{2\uppi C(0,0){\sigma }^{3}}{\sqrt{(2Dt+{\sigma }^{2})}}\left\{\sqrt{\displaystyle\frac{\uppi (2Dt+{\sigma }^{2})}{2}}{\rm{erf}}\left(\displaystyle\frac{R}{\sqrt{(4Dt+2{\sigma }^{2})}}\right)\right.\\\left.\qquad\quad-R\,{\rm{exp}}\left[-\displaystyle\frac{{R}^{2}}{(4Dt+2{\sigma }^{2})}\right]\right\}.\end{array}$$

Hence, as the ratio *M*(*t*)/*M*(0) can be determined experimentally (using equation (4)), *D* can be derived using equation (5), provided *σ* and *R* are known. If we assume that the distance that light penetrates into the tissue to initiate bleaching will be comparable to the distance light penetrates to record the fluorescence as dye diffuses back into the bleached volume, then we can set *R* = *σ*. In fact, while the time course of *M*(*t*) is sensitive to values of *D* and *σ*, it is insensitive to values of *R* (Extended Data Fig. 2), so this assumption has little impact on the derived value of *D*.

In the presence of fluid flow with a velocity *v*, the integral of equation (2) to give *M*(*t*) becomes:

6 $$M(t)=2\pi C(0,0){\left[1+\frac{2Dt}{{\sigma }^{2}}\right]}^{-\frac{3}{2}}{e}^{-\frac{{v}^{2}{t}^{2}}{4Dt+2{\sigma }^{2}}}\mathop{\int }\limits_{0}^{R}{r}^{2}{\exp}\left[\frac{-({r}^{2}+2rvt)}{4Dt+2{\sigma }^{2}}\right]dr.$$

The integral cannot be solved analytically but can be evaluated numerically (Extended Data Fig. 5).

### In vitro photobleaching protocol

The experimental setup is shown in Fig. 1a. Light from a 488-nm laser diode (Doric Lenses) was passed through a 200-μm optical fiber (Doric Lenses) into an agarose gel brain phantom (see ‘Preparation of agarose gel brain phantoms’) containing FITC-dextran (25 mg ml^−1^; Merck Life Science UK). The power at the tip of the optical fiber was measured to be 1.3 mW. Following a 30-s period of photobleaching at 20 °C, controlled by an electronic shutter triggered once every hour, the recovery of fluorescence was recorded using an LED for excitation (465-nm wavelength) and a photoreceiver (New Focus) with a 500–540-nm-wavelength Mini Cube filter) (Doric Lenses). The signal was amplified by a lock-in amplifier (Stanford Research Systems), operating at 125 Hz and stored on a computer. All photometry data were recorded with the software Doric Neuroscience Studio (v.5.4.1.23, Doric Lenses).

### In vivo photobleaching protocol

An identical setup was used for the in vivo experiments but with the 200-μm optical fiber being implanted into the frontal cortex of a male C57BL/6J mouse with coordinates: medial–lateral (ML) −1.00 mm, anterior–posterior (AP) 2.22 mm, dorsal–ventral (DV) −2.00 mm and a guide cannula being implanted in the CPu (coordinates: ML −2.55 mm, AP −0.58 mm, DV −3.00 mm) for injection of the 4 kDa FITC-dextran. At the start of the experiment, 4 kDa FITC-dextran was injected into the CPu (25 mg ml^−1^ in saline; 0.1 μl min^−1^ over 100 min), with injections being made (with different animals) throughout the 24-h cycle. The dye took about 2 h to be measurable in the frontal cortex, where it reached a peak about 6–7 h after injection (Extended Data Fig. 4a). Thereafter, there was a slow decline in baseline intensity (~6% per hour), which was corrected for by fitting the baseline to a least-squares cubic spline curve. After ~6 h, the recovery of fluorescence following photobleaching was recorded every hour for up to 24 h.

### Measurement of the distribution of bleached dye in agarose gels and the brain

The experimental setup used to measure the distribution of bleached dye from the optical fiber in both agarose gels and the brain is shown in Extended Data Fig. 1a. A brain slice (800 μm) or sheet (800 μm) of an agarose gel brain phantom (see ‘Preparation of agarose gel brain phantoms’) containing FITC-dextran was sandwiched between two 500-μm blocks of clear agarose (0.5% w/v). (The purpose of the blocks of clear agarose was to eliminate internal reflection at the gel–air interfaces which would have existed in their absence, potentially artefactually increasing the spread of light, particularly along the axial direction of the fiber.) An optical fiber (diameter 200 μm) was inserted into the central gel or brain slice and an image taken of the light distribution of a 488-nm laser diode at an intensity which avoided complete bleaching at the center of the distribution. The image was digitized and fit to a hemispherical Gaussian distribution (Extended Data Fig. 1b). To account for the small spread of the dye during the 30-s bleaching, equation (2) was integrated over 30 s and this distribution was fit to a Gaussian. This small correction never exceeded 8% (Extended Data Fig. 1c).

### Preparation of agarose gel brain phantoms

Brain phantom gels, to mimic the optical scattering and absorbance of brain tissue, were composed^22^ of 1% agarose (Sigma-Aldrich A9539) in phosphate-buffered saline (10 mM phosphate buffer, 2.7 mM KCl and 137 mM NaCl, pH 7.4; Sigma-Aldrich P4417) with 8% dried skimmed milk powder (Sigma-Aldrich 70166) and 0.1% Indian ink (Winsor and Newton 1010754). For validation of the method, 0.3 mg ml^−1^ of FITC-dextran (molecular weights 4, 10 and 70 kDa) (Sigma-Aldrich 46944, FD10S and 46945, respectively) was added to the brain phantom gel.

### Direct measurement of diffusion coefficients in agarose gel brain phantoms

Accurate values of the diffusion coefficients of the FITC-dextran molecules were determined by measuring the efflux of the fluorescent dye from a sheet of agarose gel of known thickness *L*. If, at *t* = 0, a molecule has a uniform concentration of *C*_0_ in a membrane of thickness *L* and if the membrane is bounded on one side (at *x* = 0) by an impermeable barrier, then as the molecule diffuses out of the membrane across the boundary *x* = *L*, the concentration across the membrane as a function of time is given by^33^:

7 $$C(x,t)=\frac{4\,{C}_{0}}{\pi }\mathop{\sum }\limits_{n=0}^{\infty }\frac{{(-1)}^{n}}{2n+1}{\rm{exp}}\left(-\frac{D{(2n+1)}^{2}{\uppi }^{2}t}{4{L}^{2}}\right){\rm{cos}}\frac{(2n+1)\uppi x}{2L}$$

Because of the cosine term, for values of *x* that are small compared to *L* (~20% or less), *C*(*x*,*t*) is very insensitive to *x*. Consequently, if the concentration can be measured close to the impermeable barrier (that is, close to *x* = 0), then the time course provides an accurate measurement of *D*, provided only that *L* is known.

We constructed 1-mm sheets of 1% agarose gel brain phantoms containing a chosen molecular weight of FITC-dextran (concentration 25 mg ml^−1^), bounded on one side by a glass slide and the other being exposed to a stirred solution of phosphate-buffered saline at a constant temperature (20 °C) containing the same concentrations of milk solids (8%) and India ink (0.1%). A 200-μm optical fiber was inserted immediately adjacent to the impermeable glass slide (so that *x/L* = 0.1) (Extended Data Fig. 3).

### Protocol for measuring brain clearance

For the experiments used to measure brain clearance, a similar experimental arrangement to that described above for bleaching was used (Fig. 1a), with the same coordinates for the CPu injection and cortical recording. In these experiments, however, we injected a much smaller volume of dye (0.5 μl at 5 mg ml^−1^ over 10 min) into the CPu and used a smaller dye (AF488) to speed up the dye movement and allow a complete time course to be recorded. After injection, the cannula was capped and the fluorescent intensity recorded in the cortex over several hours. We assumed that the dye spread according to equation (8) (see Fig. 2 for experimental verification and also Extended Data Fig. 6) but where *σ* is now the characteristic width of the initial Gaussian distribution of dye, rather than the width of the bleached dye, as was the case for the bleaching experiments. To account for the loss of dye due to brain clearance, the equation was multiplied by a term $(1-\frac{t}{t+\tau })$, where *τ* is the half time for clearance, giving:

8 $$C^{\prime} (r,t)=C(0,0)\left(1-\frac{t}{t+\tau }\right)\left\{{\left[1+\frac{2Dt}{{\sigma }^{2}}\right]}^{-\frac{3}{2}}\left[{\rm{exp}}\left(\frac{-{r}^{2}}{4Dt+2{\sigma }^{2}}\right)\right]\right\},$$

where $C^{\prime} (r,t)$ is the concentration when clearance is present. The percentage clearance can be calculated from the ratio of the concentrations given by equations (2) and (8):

9 $${\rm{Clearance}}\,(\%)\,=\,\left[1-\frac{C^{\prime} (r,t)}{C(r,t)}\right]\times 100=\frac{t}{t+\tau }\times 100$$

In many cases, the distance *r* between the optical fiber and the cannula could be measured postmortem but, when this was not available, the calculated distance (3.335 mm) between the two sets of coordinates was used. The average of the measured distances was 3.368 ± 0.064 mm (mean ± s.e.m.; *n* = 15).

For the anesthesia experiments, mice were injected with either an anesthetic (see ‘Anesthesia’) or saline, 1 week apart and in random order. For the sleep experiments, mice were sleep deprived for 5 h and then allowed to sleep (Extended Data Fig. 7). Recordings were made either during the wake period (for 5 h) or during the recovery sleep period, starting at the first sleep episode. These recordings were made on the same animal, 1 week apart and again in random order.

### Calibration of fluorescent intensity

The observed fluorescent intensity was converted to concentration using the data shown in Supplementary Fig. 1. For both the bleaching experiments and clearance experiments, there were linear relationships between fluorescent intensity and dye concentration. For the bleaching experiments, this was confirmed by measuring fluorescent intensity in solution as a function of concentration of 4 kDa FITC-dextran (Supplementary Fig. 1). The solution was that used to prepare the brain phantom gels (see ‘Preparation of agarose gel brain phantoms’). For the clearance experiments, fluorescence was measured either from solutions or from brain slices which had been incubated in different concentrations of dye (Supplementary Fig. 1) and imaged as described below for the histology experiments (Fig. 3).

### Mice

All experiments were performed in accordance with the UK Home Office Animal Procedures Act (1986) and all procedures were approved by the Imperial College Ethical Review Committee. Mice used in the experiments were adult male C57/BL6 mice (3–7 months old). Mice were maintained on a 12 h:12 h, light:dark cycle at constant temperature (20 °C) and humidity (50%) with ad libitum food and water. All measurements were made on mice in their home cage.

### Stereotaxic surgery

Mice were anesthetized with 2% isoflurane in oxygen by inhalation and received buprenorphine injection (0.1 mg kg^−1^ subcutaneous (s.c.)) and carprofen (5 mg kg^−1^ s.c.) and placed in a stereotaxic frame (Angle Two, Leica Microsystems) on a heat mat (ThermoStar Homeothermic Monitoring System, RDW Life Science) at 36.5 °C. Mice were implanted with two miniature screw electrodes (+1.5 mm Bregma, +1.5 mm midline; −2.0 mm Bregma, +1.5 mm midline—reference electrode) with two EMG wires (AS634, Cooner Wire). The EMG electrodes were inserted between the neck musculature. A multipin plug for an EEG–EMG device (see ‘EEG/EMG recording and sleep scoring’) was affixed to the skull with Orthodontic Resin power and Orthodontic resin liquid (TOC Dental). Mice were also implanted with a 200 μm optical fiber (Doric Lenses) in the frontal cortex (coordinates: ML −1.00 mm, AP 2.22 mm, DV −2.00 mm) and a guide cannula for delivering the FITC-dextran or AF488 into the CPu (coordinates: ML −2.55 mm, AP −0.58 mm, DV −3.00 mm). Mice were allowed to recover from surgery for at least 1 week before any experiments were performed.

### Anesthesia

For the experiments during anesthesia, mice were anesthetized (i.p.) with 200 μg kg^−1^ (60 μg ml^−1^) dexmedetomidine (Orion Parma), 100 mg kg^−1^ (20 mg ml^−1^) ketamine (Zeotis) with 20 mg kg^−1^ (4 mg ml^−1^) xylazine (Dechra) or 50 mg kg^−1^ (10 mg ml^−1^) pentobarbital (Animalcare), and kept on a heat mat (ThermoStar Homeothermic Monitoring System, RDW Life Science) at 36.5 °C. Control injections were with saline.

### EEG/EMG recording and sleep scoring

EEG and EMG signals were recorded using a miniature datalogger attached to the skull^34^. The data were downloaded and waveforms visualized using MATLAB (MathWorks). The EEG signals were high-pass filtered (0.5 Hz, −3 dB) using a digital filter and the EMG was band-pass filtered between 1 and 50 Hz (−3 dB). Power in the delta (1–4 Hz), theta (5–10 Hz) bands and theta to delta band ratio were calculated, along with the root-mean-square value of the EMG signal (averaged over a bin size of 5 s). All of these data were used to define the vigilance states of Wake, NREM sleep and rapid-eye-movement (REM) sleep, initially by an automatic script using a probability-based algorithm and Gaussian Mixture Model (ʻCode Availabilityʼ). The sensitivity and specificity when compared to experienced human sleep scorers were very high (see below). Nonetheless, after automatic scoring, each vigilance state was then screened and confirmed manually afterwards.

**Table:** 

	Scorer 1			Scorer 2		
	Wake	NREM	REM	Wake	NREM	REM
Sensitivity	0.91	0.97	0.91	0.94	0.93	0.95
Specificity	0.98	0.92	0.99	0.96	0.96	0.98

### Histology experiments

At a chosen time following dye injection into the CPu, mice were killed and their brain taken by dissection and frozen immediately in liquid pentane on dry ice. The brain was then embedded in OCT embedding matrix (CellPath) and kept frozen. Next, the brain was sliced in 60-μm coronal sections using a cryostat (CryoStar NX70, Thermo Fisher Scientific), then immediately dried and mounted on slides using DPX mountant (06522, Sigma-Aldrich). The coronal sections were imaged with a widefield microscope and Zeiss Zen Pro software (Axio Observer, Carl Zeiss) at a magnification of ×5. The average intensity of each slice was measured using ImageJ and the mean intensity in groups of four along the anterior–posterior distance was calculated. The data, when plotted against the anterior–posterior distance from the site of injection, were fitted to Gaussian curves, with variable width, amplitude, baseline and position.

### Quantification and statistical analysis

All quantitative results are quoted as means ± 95% confidence intervals or means ± s.e.m. Normality was confirmed using the Kolmogorov–Smirnov test. Comparisons were made using estimation statistics and one-way or two-way analysis of variance (ANOVA). Confidence intervals and sampling distributions (that is, distributions of likelihood) were calculated using bias-corrected and accelerated bootstrapping^35^. The sampling distributions were calculated using 5,000 bootstrap samples. Data collection and analysis were generally not performed blind to the conditions of the experiments. However, the automatic sleep-scoring algorithm was done blind and the vigilance states then checked manually. No statistical methods were used to predetermine sample sizes but our sample sizes are similar to those reported in previous publications^3,4,12^.

### Data exclusions

For the diffusion coefficient measurements, bleaching recordings that could not be fitted by the custom curve-fitting algorithm were excluded. For the photometry recordings, poor fits to the theoretical curves were excluded and recordings where one of the paired recordings (saline or anesthetic; or sleep and wake) was not successful. For the histology experiments, brain sections that were substantially damaged were excluded from the quantitative analysis.

### Reporting summary

Further information on research design is available in the Nature Portfolio Reporting Summary linked to this article.

## Online content

Any methods, additional references, Nature Portfolio reporting summaries, source data, extended data, supplementary information, acknowledgements, peer review information; details of author contributions and competing interests; and statements of data and code availability are available at 10.1038/s41593-024-01638-y.

## Supplementary information

Supplementary Information Supplementary Fig. 1.

Reporting Summary

## Source data

Source Data Fig. 1 Statistical source data.

Source Data Fig. 2 Statistical source data.

Source Data Fig. 3 Statistical source data.

Source Data Extended Data Fig. 1 Statistical source data.

Source Data Extended Data Fig. 3 Statistical source data.

Source Data Extended Data Fig. 4 Statistical source data.

Source Data Extended Data Fig. 5 Statistical source data.

Source Data Extended Data Fig. 7 Statistical source data.

Source Data Extended Data Fig. 8 Statistical source data.

Source Data Extended Data Fig. 9 Statistical source data.

## Figures and Tables

**Extended Data Table 1:**
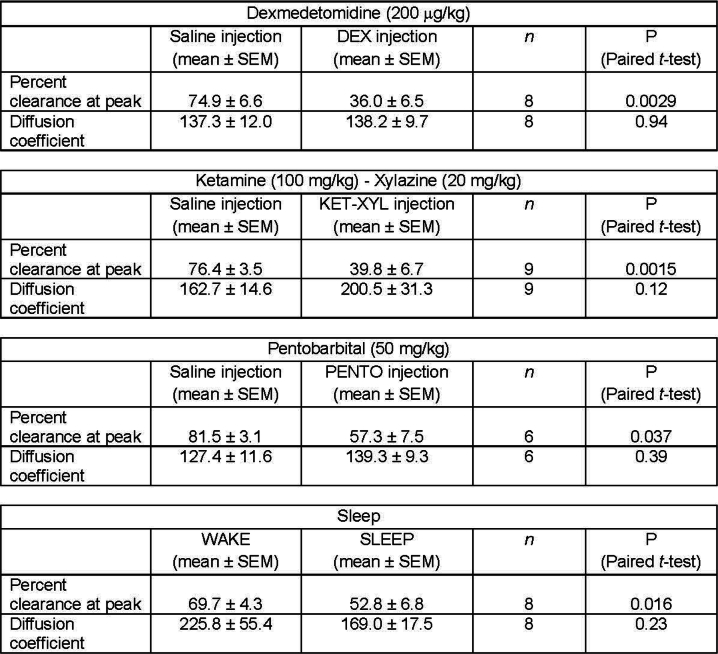
Summary of percentage clearances and diffusion coefficients

Summary of percentage clearances at the peak photometry signal (see Fig. 2h-k) and diffusion coefficients for the different vigilance states as means ± SEMs. The statistical test used was a two-tailed paired *t*-test.

## Data Availability

All source data for the main figures and Extended Data figures are available on figshare at 10.6084/m9.figshare.25483339 (ref. ^36^). Source data are provided with this paper.

## Code availability

The MATLAB script for automatic sleep scoring is available on figshare at 10.6084/m9.figshare.25483339 (ref. ^36^).
