# Author Correction: Brain clearance is reduced during sleep and anesthesia

**DOI:** 10.1038/s41593-024-01698-0

**Published:** 2024-06-14

**Authors:** Andawei Miao, Tianyuan Luo, Bryan Hsieh, Christopher J. Edge, Morgan Gridley, Ryan Tak Chun Wong, Timothy G. Constandinou, William Wisden, Nicholas P. Franks

**Affiliations:** 1 Department of Life Sciences, Imperial College London, South Kensington, London, UK; 2 UK Dementia Research Institute, Imperial College London, London, UK; 3 Centre for Doctoral Training and Centre for Neurotechnology, Imperial College London, London, UK; 4 Department of Electrical and Electronic Engineering and UK Dementia Research Institute, Care Research & Technology, Imperial College London, London, UK; 5 Present Address: Department of Anesthesiology, Affiliated Hospital of Zunyi Medical University, Zunyi, China; 6 Present Address: Guizhou Key Laboratory of Anesthesia and Organ Protection, Zunyi Medical University, Zunyi, China

**Keywords:** Sleep, Biophysics

Correction to: *Nature Neuroscience* 10.1038/s41593-024-01638-y, published online 13 May 2024

In the version of the article initially published, one column in the source data for Fig. 3b (Mean Wake 3h) was incorrect and an amended version has now been uploaded online.

