# Supplementary material for: Brain clearance is reduced during sleep and anesthesia
Source: Nat Neurosci. 2024 May 13;27(6):1046–50. doi: 10.1038/s41593-024-01638-y (PMC11156584; doi:10.1038/s41593-024-01638-y)
Supplement: Supplementary file 1 — Supplementary Fig. 1. [file 41593_2024_1638_MOESM1_ESM.pdf]

nature neuroscience

Brief Communication https://doi.org/10.1038/s41593-024-01638-y

Brain clearance is reduced during sleep and anesthesia

In the format provided by the authors and unedited

Supplementary Data Fig. 1. Linear relationships existed between fluorescent intensity and concentration. The fluorescent intensity recorded using the optical fiber was linearly related to the concentration of **a**, AF488 (n = 5 independent experiments) and **b**, 4k-FITC-dextran (n = 5 independent experiments) in solution. This calibration was made using the same solution used to prepare the brain phantoms gels (see Methods). Linearity was also observed with imaged brain slices that had been incubated with varying concentrations of either **c**, AF488 (n = 8 independent experiments) of **d**, 4k-FITC-dextran (n = 6 independent experiments). Where error bars (SEM) are not shown they were smaller than the size of the symbol.

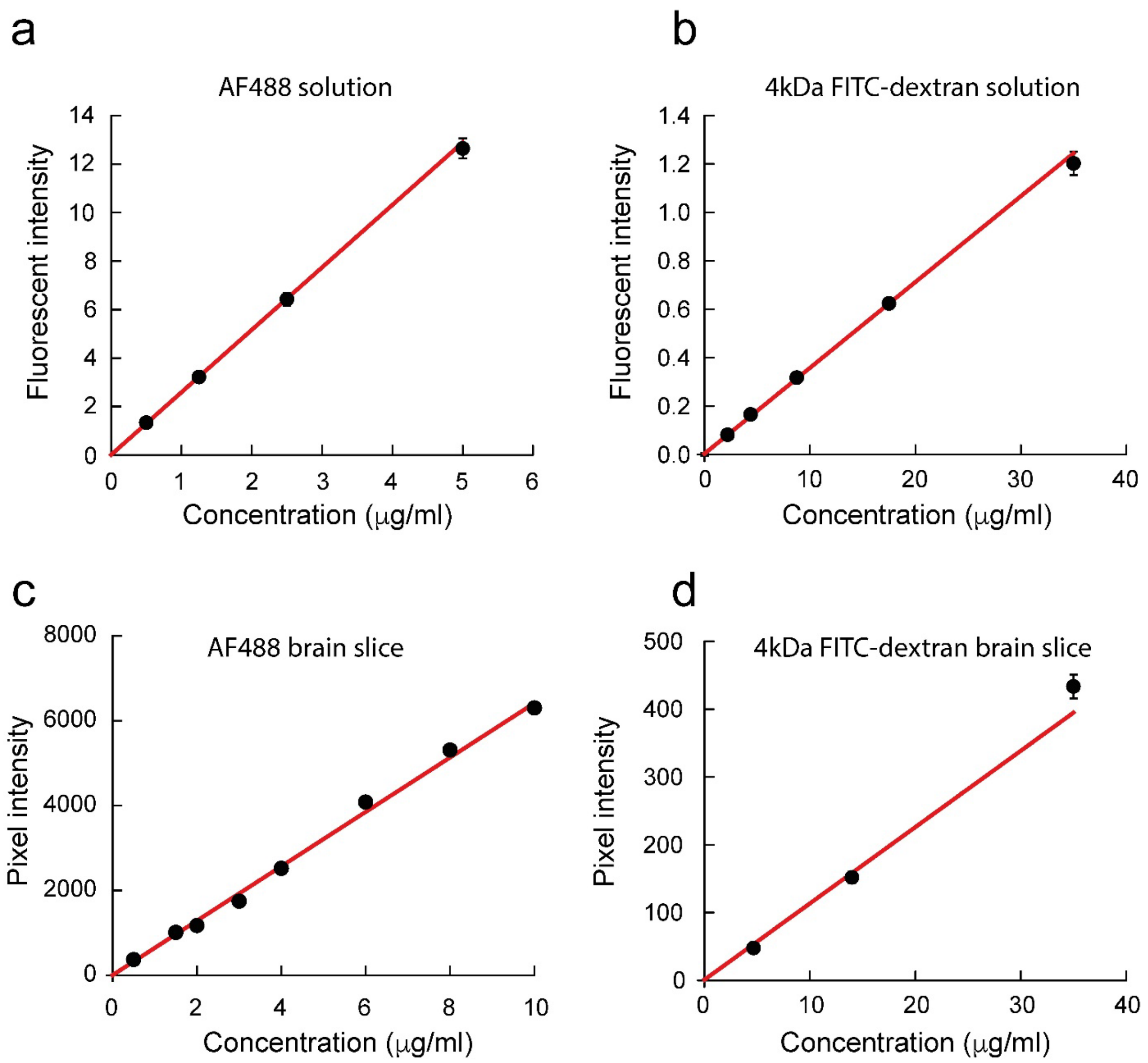

a
AF488 solution
Fluorescent intensity
Concentration (μg/ml)
b
4kDa FITC-dextran solution
Fluorescent intensity
Concentration (μg/ml)
c
AF488 brain slice
Pixel intensity
Concentration (μg/ml)
d
4kDa FITC-dextran brain slice
Pixel intensity
Concentration (μg/ml)
