# Supplementary material for: Brain clearance is reduced during sleep and anesthesia
Source: Nat Neurosci. 2024 May 13;27(6):1046–50. doi: 10.1038/s41593-024-01638-y (PMC11156584; doi:10.1038/s41593-024-01638-y)
Supplement: Supplementary file 2 — Reporting Summary [file 41593_2024_1638_MOESM2_ESM.pdf]

Corresponding author(s): Nicholas P. Franks

Last updated by author(s): Mar 27, 2024

Reporting Summary

Nature Portfolio wishes to improve the reproducibility of the work that we publish. This form provides structure for consistency and transparency in reporting. For further information on Nature Portfolio policies, see our Editorial Policies and the Editorial Policy Checklist.

Statistics

For all statistical analyses, confirm that the following items are present in the figure legend, table legend, main text, or Methods section.

n/a	Confirmed	
☐	☒	The exact sample size (*n*) for each experimental group/condition, given as a discrete number and unit of measurement
☐	☒	A statement on whether measurements were taken from distinct samples or whether the same sample was measured repeatedly
☐	☒	The statistical test(s) used AND whether they are one- or two-sided<br>*Only common tests should be described solely by name; describe more complex techniques in the Methods section.*
☐	☒	A description of all covariates tested
☐	☒	A description of any assumptions or corrections, such as tests of normality and adjustment for multiple comparisons
☐	☒	A full description of the statistical parameters including central tendency (e.g. means) or other basic estimates (e.g. regression coefficient) AND variation (e.g. standard deviation) or associated estimates of uncertainty (e.g. confidence intervals)
☐	☒	For null hypothesis testing, the test statistic (e.g. *F*, *t*, *r*) with confidence intervals, effect sizes, degrees of freedom and *P* value noted<br>*Give P values as exact values whenever suitable.*
☒	☐	For Bayesian analysis, information on the choice of priors and Markov chain Monte Carlo settings
☒	☐	For hierarchical and complex designs, identification of the appropriate level for tests and full reporting of outcomes
☒	☐	Estimates of effect sizes (e.g. Cohen's *d*, Pearson's *r*), indicating how they were calculated

Our web collection on statistics for biologists contains articles on many of the points above.

Software and code

Policy information about availability of computer code

Data collection	EEG/EMG data was collected with a customized portable recording device (Hsieh, B. et al. 2019). Photometry data was collected with Doric Lenses photometry system (Doric Lenses, Quebec Canada) and recored with the software Doric Neuroscience Studio (version 5.4.1.23, Doric Lenses, Quebec Canada). Histology data was collected with a widefield microscope Zeiss Axio Observer 3 with Zeiss Zen Pro software (version 3.8, Carl Zeiss NY U.S.)
Data analysis	Automatic sleep scoring based on EEG/EMG and photo-recovery curve fitting were carried out simultaneously with a customized script performed in software Matlab (The MathWorks Inc, Natick, Massachusetts, USA, version R2024a, 24.1). Vigilance states were checked manually. The script and related documentation is included in the Code and Software Submission. Statistical calculations were made using the online resource https://www.estimationstats.com/#/ (Ho, J. et al. 2019), Matlab (The MathWorks Inc, Natick, Massachusetts, USA, version R2024a, 24.1 or OriginPro (OriginLab Corporation, Massachusetts, USA, version 9.8.0.200). Figures were prepared with Adobe Illustrator (version 26.5.2). Mean pixel intensity for histological data and preparation of representative images were performed with FIJI ImageJ (version 1.54f).

For manuscripts utilizing custom algorithms or software that are central to the research but not yet described in published literature, software must be made available to editors and reviewers. We strongly encourage code deposition in a community repository (e.g. GitHub). See the Nature Portfolio guidelines for submitting code & software for further information.

Data

Policy information about availability of data

All manuscripts must include a data availability statement. This statement should provide the following information, where applicable:

- Accession codes, unique identifiers, or web links for publicly available datasets
- A description of any restrictions on data availability
- For clinical datasets or third party data, please ensure that the statement adheres to our policy

All data are available from the corresponding authors on request.

Research involving human participants, their data, or biological material

Policy information about studies with human participants or human data. See also policy information about sex, gender (identity/presentation), and sexual orientation and race, ethnicity and racism.

Reporting on sex and gender	N.A.
Reporting on race, ethnicity, or other socially relevant groupings	N.A.
Population characteristics	N.A.
Recruitment	N.A.
Ethics oversight	N.A.

Note that full information on the approval of the study protocol must also be provided in the manuscript.

Field-specific reporting

Please select the one below that is the best fit for your research. If you are not sure, read the appropriate sections before making your selection.

☒ Life sciences ☐ Behavioural & social sciences ☐ Ecological, evolutionary & environmental sciences

For a reference copy of the document with all sections, see nature.com/documents/nr-reporting-summary-flat.pdf

Life sciences study design

All studies must disclose on these points even when the disclosure is negative.

Sample size	No statistical methods were used to pre-determine sample sizes but our sample sizes are similar to those reported in previous publications (Iliff et al.,2012 PMID: 22896675; Xie et al., 2013 PMID: 24136970; Mestre et al., 2018 PMID: 30451853).
Data exclusions	For the diffusion coefficient measurements, bleaching recordings that could not be fitted by the custom curve-fitting algorithm were excluded. For the photometry recordings, poor fits to the theoretical curves were excluded, and recordings where one of the paired recordings (either saline or anesthetic, or sleep and wake) was not successful. For the histology experiments, brain sections that were significantly damaged were excluded from the quantitative analysis.
Replication	The main method of photo-bleaching and recovery has been validated independently in vitro with FITC-dextran at different molecular weights. In vivo photo-bleaching and recovery experiment were carried out in a replication of 24 animals. Each animal were recorded during multiple experiments each contains multiple bleach/recovery successions. For in vivo photometry experiments, at least 6 animals were tested for each anesthetics and vigilant states. Multiple measurements were made for each experimental conditions. Histology images shows in Figures were repeated in at least three mice. All above mentioned attempts at replication were successful.
Randomization	Selection of animals from the stock cohort were randomized. In vivo photo-bleaching and recovery experiments were started at random time of the day. For the anesthesia experiments, mice were injected with either an anesthetic or saline in random order. For the sleep experiments, recordings were made on the same animal, one week apart, in random order.
Blinding	Data collection and analysis were generally not performed blind to the conditions of the experiments. However, the automatic sleep-scoring algorithm was done blind, and the vigilance states then checked manually.

Reporting for specific materials, systems and methods

We require information from authors about some types of materials, experimental systems and methods used in many studies. Here, indicate whether each material, system or method listed is relevant to your study. If you are not sure if a list item applies to your research, read the appropriate section before selecting a response.

Materials & experimental systems

n/a	Involved in the study
☒	☐ Antibodies
☒	☐ Eukaryotic cell lines
☒	☐ Palaeontology and archaeology
☐	☒ Animals and other organisms
☒	☐ Clinical data
☒	☐ Dual use research of concern
☒	☐ Plants

Methods

n/a	Involved in the study
☒	☐ ChIP-seq
☒	☐ Flow cytometry
☒	☐ MRI-based neuroimaging

Animals and other research organisms

Policy information about studies involving animals; ARRIVE guidelines recommended for reporting animal research, and Sex and Gender in Research

Laboratory animals	C57BL6j (Jackson laboratory), male, aged between 3-7 months
Wild animals	No wild animals were used in the research
Reporting on sex	Metabolite clearance and the function of sleep are not considered as a sex dimorphism according to the current literature. Therefore only male mice were used in this research. We do not expect that the results we are reporting are sex-dependent.
Field-collected samples	The study did not involve samples collected from the field.
Ethics oversight	All experiments were performed in accordance with the United Kingdom Animal Scientific Procedures Act 1986 under personal and project licenses granted by the United Kingdom Home Office. Ethical approval was provided by the Ethical Review Panel at the Imperial College London

Note that full information on the approval of the study protocol must also be provided in the manuscript.

Plants

Seed stocks	N.A.
Novel plant genotypes	N.A.
Authentication	N.A.
